# Paliperidonium nitrate

**DOI:** 10.1107/S160053681203841X

**Published:** 2012-09-15

**Authors:** Jingshui Ge, Yang-Hui Luo

**Affiliations:** a School of Pharmacy, Tongji Medical College, Huazhong University of Science and Technology, Wuhan, People’s Republic of China; b College of Chemistry and Chemical Engineering, Southeast University, Nanjing 210096, People’s Republic of China

## Abstract

In the title mol­ecular salt (systematic name: 3-{2-[4-(6-fluoro-1,2-benzoxazol-3-yl)piperidin-1-yl]eth­yl}-9-hy­droxy-2-methyl-1,6,7,8,9,9a-hexa­hydro­pyrido[1,2-*a*]pyrimidin-4-one nitrate), C_23_H_29_FN_4_O_3_^+^·NO_3_^−^, the piperidine ring displays a chair conformation and its N atom is protonated; the N—H bond is in an axial orientation. The ring bearing the hy­droxy group exhibits a half-chair conformation. The hy­droxy group as well as the adjacent methyl­ene group are disordered over two sets of sites in a 0.823 (5):0.177 (5) ratio. In the crystal, O—H⋯N, O—H⋯O, N—H⋯O and N—H⋯N hydrogen bonds connect the components into a three-dimensional network.

## Related literature

For polymorphism of pharmaceutical materials, see: Luo *et al.* (2012 ▶). For background to the anti-psychotic drug paliperidone, see: Spina & Crupi (2011 ▶).
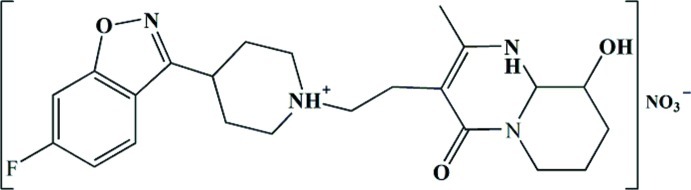


## Experimental

### 

#### Crystal data

C_23_H_29_FN_4_O_3_^+^·NO_3_^−^
*M* *_r_* = 490.51
Monoclinic, 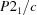

*a* = 8.3642 (8) Å
*b* = 22.032 (2) Å
*c* = 12.4485 (13) Å
β = 92.311 (3)°
*V* = 2292.1 (4) Å^3^
*Z* = 4
Mo *K*α radiation
μ = 0.11 mm^−1^
*T* = 293 K
0.30 × 0.25 × 0.24 mm

#### Data collection

Rigaku SCXmini diffractometer
Absorption correction: multi-scan (*CrystalClear*; Rigaku, 2005 ▶) *T*_min_ = 0.968, *T*_max_ = 0.974
23571 measured reflections
5234 independent reflections
2824 reflections with *I* > 2σ(*I*)
*R*_int_ = 0.063

#### Refinement

*R*[*F* ^2^ > 2σ(*F* ^2^)] = 0.063
*wR*(*F* ^2^) = 0.178
*S* = 1.06
5234 reflections
353 parameters
H-atom parameters constrained
Δρ_max_ = 0.42 e Å^−3^
Δρ_min_ = −0.45 e Å^−3^

### 

Data collection: *CrystalClear* (Rigaku, 2005 ▶); cell refinement: *CrystalClear*; data reduction: *CrystalClear*; program(s) used to solve structure: *SHELXS97* (Sheldrick, 2008 ▶); program(s) used to refine structure: *SHELXL97* (Sheldrick, 2008 ▶); molecular graphics: *DIAMOND* (Brandenburg & Putz, 2005 ▶); software used to prepare material for publication: *SHELXL97*.

## Supplementary Material

Crystal structure: contains datablock(s) I, New_Global_Publ_Block. DOI: 10.1107/S160053681203841X/hb6895sup1.cif

Structure factors: contains datablock(s) I. DOI: 10.1107/S160053681203841X/hb6895Isup2.hkl

Additional supplementary materials:  crystallographic information; 3D view; checkCIF report

## Figures and Tables

**Table 1:** Hydrogen-bond geometry (Å, °)

*D*—H⋯*A*	*D*—H	H⋯*A*	*D*⋯*A*	*D*—H⋯*A*
N2—H2⋯O4′	0.91	1.94	2.823 (18)	164
N2—H2⋯O5	0.91	2.15	3.028 (12)	161
N2—H2⋯O6	0.91	2.23	2.999 (9)	142
N2—H2⋯N5	0.91	2.56	3.465 (3)	170
N2—H2⋯O5′	0.91	2.58	3.36 (3)	144
O3′—H3′⋯N1^i^	0.82	2.30	3.117 (18)	169
O3—H3⋯O5^i^	0.82	2.11	2.915 (12)	168
O3—H3⋯O4′^i^	0.82	2.27	3.064 (19)	163
O3—H3⋯O6′^i^	0.82	2.30	2.93 (4)	134
O3—H3⋯O4^i^	0.82	2.60	3.187 (16)	130
O3—H3⋯N5^i^	0.82	2.67	3.423 (4)	153

Symmetry code: (i) 
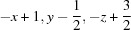
.

**Fig. 1.**
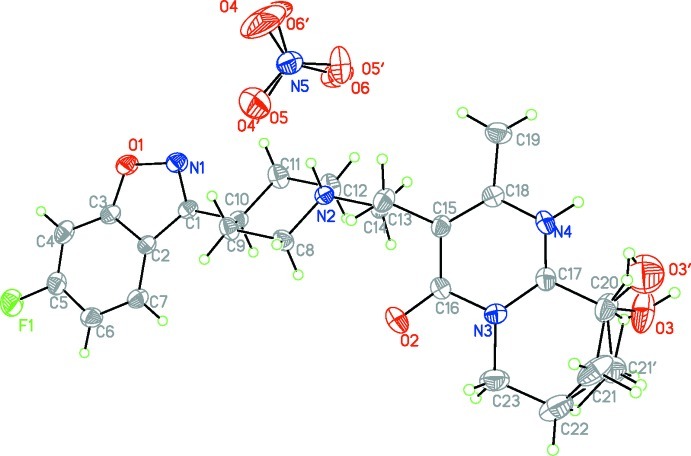
The molecular structure of the title compound with displacement ellipsoids drawn at the 30% probability level.

**Fig. 2.**
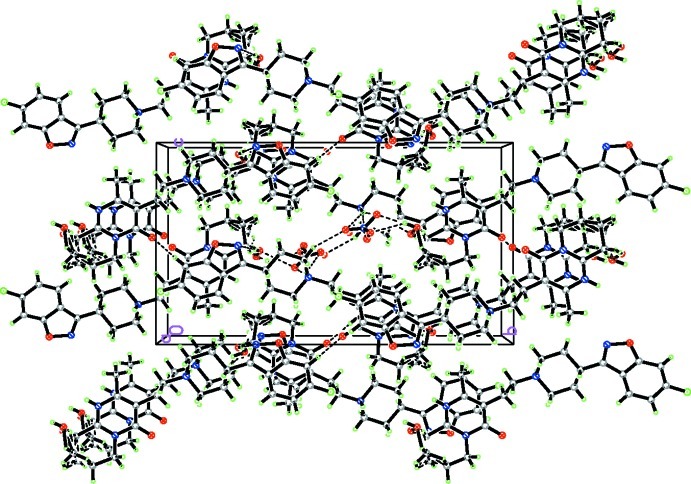
A packing view showing the three dimensionnal network. Intermolecular hydrogen bonds are shown as dashed lines.

## supplementary crystallographic information

### Comment

In recent years, the studies of polymorphism for pharmaceutical ingredients have received much attention, different polymorphs can affect shelf life,durability, solubility, as well as bioavailability and manufacturing of a drug (Luo *et al.*,2012). Paliperidone or 9-hydroxyrisperidone, is one of the most recently available atypical antipsychotics (Spina & Crupi, 2011). It is a benzisoxazole derivative and the major active metabolite of risperidone, a widely used atypical antipsychotic approved for the treatment of schizophrenia and other psychiatric disorders. In view of the importance of the polymorphs of paliperidone, we reported here a pesudo-polymorphism of paliperidone: paliperidonium nitrate.

The asymmetric unit of the title compound consisting of a paliperidone cation and a nitrate ion (Fig. 1) The piperidine ring of the paliperidone cation displays a chair conformation with the nitrogen atoms charged with a hydrion, while the ring which bears the hydroxy group exhibits a half-chair conformation. The hydroxy group as well as the neighbour carbon atom (C21), are disordered over two positions, both with site occupancy factors of 0.82263 and 0.17737.

In the crystal, inteicate classical O—H···N, O—H···O, N—H···O and N—H···N hydrogen bonds between the paliperidone cation and the nitrate ion are observed, which connect the molecules into complex structure.(Fig. 2).

### Experimental

The title compound are provided by Changzhou Siyao Pharmaceuticals Co. Ltd (Changzhou, China). Yellow blocks were obstained by slow evaporation of a methanol solution.

### Refinement

All H atoms attached to C atoms, N atoms and O atoms were fixed geometrically and treated as riding with C—H = 0.93 Å (CH), C—H = 0.97 Å (CH_2_), C—H = 0.96 Å (CH_3_), N—H = 0.86 Å and O—H = 0.82 Å with *U*_iso_(H) =1.2*U*_eq_(CH, CH_2_ and NH) and *U*_iso_(H) = 1.5*U*_eq_(CH_3_ and OH).

### Crystal data

**Table:**

C_23_H_29_FN_4_O_3_^+^·NO_3_^−^	*F*(000) = 1036
*M**_r_* = 490.51	*D*_x_ = 1.421 Mg m^−^^3^
Monoclinic, *P*2_1_/*c*	Mo *K*α radiation, λ = 0.71075 Å
Hall symbol: -P 2ybc	Cell parameters from 5234 reflections
*a* = 8.3642 (8) Å	θ = 3.0–27.5°
*b* = 22.032 (2) Å	µ = 0.11 mm^−^^1^
*c* = 12.4485 (13) Å	*T* = 293 K
β = 92.311 (3)°	Block, yellow
*V* = 2292.1 (4) Å^3^	0.30 × 0.25 × 0.24 mm
*Z* = 4	

### Data collection

**Table:**

Rigaku SCXmini diffractometer	5234 independent reflections
Radiation source: fine-focus sealed tube	2824 reflections with *I* > 2σ(*I*)
Graphite monochromator	*R*_int_ = 0.063
Detector resolution: 13.6612 pixels mm^-1^	θ_max_ = 27.5°, θ_min_ = 3.0°
CCD_Profile_fitting scans	*h* = −10→10
Absorption correction: multi-scan (*CrystalClear*; Rigaku, 2005)	*k* = −28→28
*T*_min_ = 0.968, *T*_max_ = 0.974	*l* = −16→16
23571 measured reflections	

### Refinement

**Table:**

Refinement on *F*^2^	Primary atom site location: structure-invariant direct methods
Least-squares matrix: full	Secondary atom site location: difference Fourier map
*R*[*F*^2^ > 2σ(*F*^2^)] = 0.063	Hydrogen site location: inferred from neighbouring sites
*wR*(*F*^2^) = 0.178	H-atom parameters constrained
*S* = 1.06	*w* = 1/[σ^2^(*F*_o_^2^) + (0.0748*P*)^2^ + 0.3819*P*] where *P* = (*F*_o_^2^ + 2*F*_c_^2^)/3
5234 reflections	(Δ/σ)_max_ = 0.003
353 parameters	Δρ_max_ = 0.42 e Å^−^^3^
0 restraints	Δρ_min_ = −0.45 e Å^−^^3^

### Special details

**Table:**

Geometry. All e.s.d.'s (except the e.s.d. in the dihedral angle between two l.s. planes) are estimated using the full covariance matrix. The cell e.s.d.'s are taken into account individually in the estimation of e.s.d.'s in distances, angles and torsion angles; correlations between e.s.d.'s in cell parameters are only used when they are defined by crystal symmetry. An approximate (isotropic) treatment of cell e.s.d.'s is used for estimating e.s.d.'s involving l.s. planes.
Refinement. Refinement of *F*^2^ against ALL reflections. The weighted *R*-factor *wR* and goodness of fit *S* are based on *F*^2^, conventional *R*-factors *R* are based on *F*, with *F* set to zero for negative *F*^2^. The threshold expression of *F*^2^ > σ(*F*^2^) is used only for calculating *R*-factors(gt) *etc*. and is not relevant to the choice of reflections for refinement. *R*-factors based on *F*^2^ are statistically about twice as large as those based on *F*, and *R*- factors based on ALL data will be even larger.

### Fractional atomic coordinates and isotropic or equivalent isotropic displacement parameters (Å^2^)

**Table:**

	*x*	*y*	*z*	*U*_iso_*/*U*_eq_	Occ. (<1)
F1	0.0565 (2)	0.98896 (7)	0.73885 (17)	0.0799 (6)	
O1	0.2639 (2)	0.84040 (8)	0.50551 (14)	0.0536 (5)	
O2	0.2927 (3)	0.47739 (9)	0.97215 (18)	0.0750 (7)	
N1	0.2679 (3)	0.77565 (10)	0.51391 (17)	0.0492 (6)	
N2	0.3289 (2)	0.57545 (8)	0.67565 (15)	0.0362 (5)	
H2	0.4299	0.5821	0.6536	0.043*	
N3	0.2952 (2)	0.37405 (9)	0.97199 (16)	0.0424 (5)	
N4	0.4064 (3)	0.32060 (9)	0.83226 (17)	0.0457 (5)	
H4A	0.4284	0.2865	0.8028	0.055*	
C1	0.1967 (3)	0.76100 (11)	0.60118 (18)	0.0375 (6)	
C2	0.1398 (3)	0.81375 (11)	0.65504 (19)	0.0367 (6)	
C3	0.1871 (3)	0.86150 (12)	0.5923 (2)	0.0432 (6)	
C4	0.1608 (3)	0.92174 (12)	0.6162 (2)	0.0517 (7)	
H4	0.1951	0.9534	0.5733	0.062*	
C5	0.0804 (3)	0.93103 (12)	0.7081 (3)	0.0523 (7)	
C6	0.0240 (3)	0.88531 (12)	0.7730 (2)	0.0519 (7)	
H6	−0.0335	0.8948	0.8332	0.062*	
C7	0.0542 (3)	0.82570 (12)	0.7472 (2)	0.0464 (7)	
H7	0.0187	0.7942	0.7898	0.056*	
C8	0.2995 (4)	0.61826 (11)	0.76569 (19)	0.0467 (7)	
H8A	0.3792	0.6116	0.8232	0.056*	
H8B	0.1951	0.6100	0.7937	0.056*	
C9	0.3062 (3)	0.68388 (11)	0.72972 (19)	0.0447 (6)	
H9A	0.4138	0.6933	0.7087	0.054*	
H9B	0.2813	0.7101	0.7894	0.054*	
C10	0.1887 (3)	0.69623 (11)	0.63561 (19)	0.0395 (6)	
H10	0.0805	0.6884	0.6596	0.047*	
C11	0.2218 (4)	0.65205 (12)	0.5461 (2)	0.0507 (7)	
H11A	0.1436	0.6581	0.4874	0.061*	
H11B	0.3268	0.6604	0.5192	0.061*	
C12	0.2153 (4)	0.58721 (12)	0.5825 (2)	0.0523 (7)	
H12A	0.1074	0.5777	0.6028	0.063*	
H12B	0.2414	0.5608	0.5233	0.063*	
C13	0.3179 (3)	0.51047 (11)	0.7099 (2)	0.0441 (6)	
H13A	0.3105	0.4851	0.6461	0.053*	
H13B	0.2200	0.5051	0.7480	0.053*	
C14	0.4572 (3)	0.48869 (11)	0.7812 (2)	0.0474 (7)	
H14A	0.5519	0.4846	0.7393	0.057*	
H14B	0.4798	0.5179	0.8380	0.057*	
C15	0.4152 (3)	0.42850 (10)	0.8288 (2)	0.0382 (6)	
C16	0.3326 (3)	0.43048 (11)	0.9267 (2)	0.0446 (6)	
C17	0.3373 (3)	0.32160 (11)	0.9231 (2)	0.0402 (6)	
C18	0.4442 (3)	0.37363 (11)	0.78350 (19)	0.0392 (6)	
C19	0.5206 (4)	0.36680 (13)	0.6777 (2)	0.0557 (7)	
H19A	0.4397	0.3679	0.6208	0.084*	
H19B	0.5762	0.3287	0.6759	0.084*	
H19C	0.5951	0.3994	0.6684	0.084*	
C20	0.3052 (4)	0.26146 (13)	0.9775 (3)	0.0589 (8)	
H20	0.4009	0.2362	0.9722	0.071*	0.85
H20'	0.1988	0.2505	0.9478	0.071*	0.15
C21	0.2807 (5)	0.2714 (2)	1.0991 (4)	0.0731 (14)	0.823 (5)
H21A	0.2492	0.2335	1.1320	0.088*	0.823 (5)
H21B	0.3801	0.2847	1.1343	0.088*	0.823 (5)
C21'	0.194 (3)	0.2599 (8)	1.0543 (16)	0.059 (5)*	0.177 (5)
H21C	0.2285	0.2300	1.1075	0.071*	0.177 (5)
H21D	0.0947	0.2451	1.0207	0.071*	0.177 (5)
C22	0.1568 (5)	0.31708 (17)	1.1125 (3)	0.0850 (11)	
H22A	0.1315	0.3197	1.1877	0.102*	
H22B	0.0604	0.3053	1.0718	0.102*	
C23	0.2119 (4)	0.37704 (15)	1.0748 (2)	0.0643 (8)	
H23A	0.2838	0.3945	1.1295	0.077*	
H23B	0.1203	0.4038	1.0658	0.077*	
O3	0.1799 (3)	0.23263 (11)	0.9224 (3)	0.0890 (13)	0.823 (5)
H3	0.2103	0.1997	0.9003	0.133*	0.823 (5)
O3'	0.391 (2)	0.2197 (8)	0.9559 (14)	0.118 (7)*	0.177 (5)
H3'	0.4856	0.2297	0.9625	0.177*	0.177 (5)
O4	0.8275 (16)	0.5916 (7)	0.5210 (7)	0.086 (2)	0.69
O5	0.6654 (13)	0.6207 (5)	0.6430 (12)	0.120 (4)	0.69
O6	0.6165 (11)	0.5413 (5)	0.5526 (7)	0.089 (3)	0.69
N5	0.7082 (3)	0.58315 (14)	0.5744 (2)	0.0584 (7)	
O4'	0.640 (2)	0.6163 (7)	0.632 (2)	0.069 (5)	0.31
O5'	0.670 (3)	0.5314 (10)	0.565 (2)	0.127 (9)	0.31
O6'	0.837 (4)	0.600 (2)	0.552 (2)	0.176 (14)	0.31

### Atomic displacement parameters (Å^2^)

**Table:**

	*U*^11^	*U*^22^	*U*^33^	*U*^12^	*U*^13^	*U*^23^
F1	0.0704 (12)	0.0454 (10)	0.1252 (17)	0.0094 (8)	0.0184 (11)	−0.0060 (10)
O1	0.0679 (13)	0.0498 (12)	0.0440 (11)	−0.0008 (9)	0.0128 (10)	0.0146 (9)
O2	0.1027 (17)	0.0433 (12)	0.0812 (15)	0.0030 (11)	0.0313 (13)	−0.0196 (11)
N1	0.0595 (15)	0.0489 (14)	0.0396 (12)	0.0013 (11)	0.0068 (11)	0.0076 (10)
N2	0.0362 (11)	0.0363 (11)	0.0364 (11)	−0.0011 (9)	0.0041 (9)	0.0015 (9)
N3	0.0479 (13)	0.0439 (13)	0.0359 (11)	−0.0028 (10)	0.0082 (9)	0.0004 (10)
N4	0.0565 (14)	0.0301 (11)	0.0508 (13)	0.0033 (10)	0.0063 (11)	−0.0068 (10)
C1	0.0384 (13)	0.0430 (14)	0.0310 (13)	−0.0021 (11)	−0.0010 (11)	0.0077 (11)
C2	0.0359 (13)	0.0370 (14)	0.0370 (13)	−0.0007 (10)	0.0003 (11)	0.0062 (11)
C3	0.0438 (15)	0.0453 (15)	0.0406 (14)	−0.0006 (12)	0.0017 (12)	0.0115 (12)
C4	0.0480 (16)	0.0374 (15)	0.069 (2)	0.0009 (12)	−0.0005 (15)	0.0176 (14)
C5	0.0422 (15)	0.0370 (15)	0.078 (2)	0.0061 (12)	0.0033 (15)	−0.0002 (14)
C6	0.0444 (16)	0.0525 (17)	0.0595 (18)	0.0051 (13)	0.0114 (14)	−0.0003 (14)
C7	0.0458 (15)	0.0452 (15)	0.0488 (16)	−0.0003 (12)	0.0084 (13)	0.0094 (13)
C8	0.0687 (18)	0.0389 (14)	0.0328 (13)	0.0043 (12)	0.0054 (13)	0.0001 (11)
C9	0.0656 (18)	0.0353 (14)	0.0327 (13)	0.0050 (12)	−0.0034 (12)	−0.0007 (11)
C10	0.0403 (14)	0.0387 (14)	0.0399 (14)	0.0021 (11)	0.0064 (11)	0.0054 (11)
C11	0.0675 (19)	0.0487 (16)	0.0347 (14)	0.0039 (13)	−0.0123 (13)	−0.0008 (12)
C12	0.0672 (19)	0.0433 (16)	0.0447 (16)	0.0039 (13)	−0.0181 (14)	−0.0020 (13)
C13	0.0469 (15)	0.0340 (14)	0.0514 (15)	−0.0026 (11)	0.0013 (13)	0.0051 (12)
C14	0.0425 (15)	0.0415 (15)	0.0582 (17)	−0.0025 (12)	0.0026 (13)	0.0104 (13)
C15	0.0396 (14)	0.0320 (13)	0.0431 (14)	−0.0002 (10)	0.0018 (11)	0.0051 (11)
C16	0.0500 (16)	0.0350 (14)	0.0496 (16)	0.0017 (12)	0.0095 (13)	−0.0043 (12)
C17	0.0397 (14)	0.0341 (14)	0.0467 (15)	0.0001 (11)	−0.0009 (12)	−0.0001 (12)
C18	0.0371 (14)	0.0431 (15)	0.0371 (13)	0.0006 (11)	−0.0014 (11)	0.0027 (12)
C19	0.0607 (18)	0.0643 (19)	0.0429 (15)	0.0016 (14)	0.0127 (14)	−0.0053 (14)
C20	0.0535 (18)	0.0423 (16)	0.081 (2)	0.0009 (14)	0.0059 (17)	0.0162 (15)
C21	0.049 (2)	0.099 (3)	0.071 (3)	−0.004 (2)	−0.004 (2)	0.054 (2)
C22	0.093 (3)	0.097 (3)	0.067 (2)	−0.015 (2)	0.025 (2)	0.022 (2)
C23	0.071 (2)	0.081 (2)	0.0419 (16)	−0.0121 (17)	0.0167 (15)	−0.0057 (15)
O3	0.0617 (19)	0.0486 (16)	0.154 (3)	−0.0114 (12)	−0.0312 (18)	0.0149 (17)
O4	0.048 (4)	0.129 (5)	0.083 (3)	−0.012 (3)	0.028 (2)	0.007 (3)
O5	0.159 (9)	0.103 (6)	0.100 (5)	−0.019 (5)	0.039 (6)	−0.024 (5)
O6	0.070 (3)	0.127 (7)	0.071 (3)	−0.037 (4)	0.016 (2)	−0.016 (3)
N5	0.0556 (19)	0.0624 (19)	0.0579 (17)	0.0019 (16)	0.0097 (15)	0.0076 (14)
O4'	0.042 (5)	0.030 (5)	0.138 (15)	0.005 (4)	0.036 (6)	−0.018 (7)
O5'	0.16 (2)	0.054 (8)	0.167 (17)	−0.032 (12)	0.039 (14)	−0.035 (8)
O6'	0.065 (14)	0.17 (2)	0.30 (4)	0.005 (12)	0.09 (2)	0.06 (3)

### Geometric parameters (Å, º)

**Table:**

F1—C5	1.350 (3)	C13—H13A	0.9700
O1—C3	1.360 (3)	C13—H13B	0.9700
O1—N1	1.431 (3)	C14—C15	1.500 (3)
O2—C16	1.231 (3)	C14—H14A	0.9700
N1—C1	1.301 (3)	C14—H14B	0.9700
N2—C12	1.492 (3)	C15—C18	1.360 (3)
N2—C8	1.493 (3)	C15—C16	1.426 (4)
N2—C13	1.498 (3)	C17—C20	1.517 (4)
N2—H2	0.9108	C18—C19	1.495 (3)
N3—C17	1.359 (3)	C19—H19A	0.9600
N3—C16	1.405 (3)	C19—H19B	0.9600
N3—C23	1.483 (3)	C19—H19C	0.9600
N4—C17	1.291 (3)	C20—O3'	1.204 (17)
N4—C18	1.360 (3)	C20—C21'	1.362 (18)
N4—H4A	0.8594	C20—O3	1.383 (4)
C1—C2	1.433 (3)	C20—C21	1.552 (5)
C1—C10	1.492 (3)	C20—H20	0.9798
C2—C3	1.378 (3)	C20—H20'	0.9803
C2—C7	1.401 (3)	C21—C22	1.459 (5)
C3—C4	1.380 (4)	C21—H21A	0.9700
C4—C5	1.365 (4)	C21—H21B	0.9700
C4—H4	0.9300	C21'—C22	1.492 (18)
C5—C6	1.386 (4)	C21'—H21C	0.9700
C6—C7	1.378 (4)	C21'—H21D	0.9700
C6—H6	0.9300	C22—C23	1.482 (4)
C7—H7	0.9300	C22—H22A	0.9700
C8—C9	1.515 (3)	C22—H22B	0.9700
C8—H8A	0.9700	C23—H23A	0.9700
C8—H8B	0.9700	C23—H23B	0.9700
C9—C10	1.523 (3)	O3—H20'	0.5247
C9—H9A	0.9700	O3—H3	0.8192
C9—H9B	0.9700	O3'—H20	0.4223
C10—C11	1.514 (3)	O3'—H3'	0.8231
C10—H10	0.9800	O4—N5	1.235 (13)
C11—C12	1.500 (3)	O5—N5	1.252 (11)
C11—H11A	0.9700	O6—N5	1.223 (10)
C11—H11B	0.9700	N5—O5'	1.19 (2)
C12—H12A	0.9700	N5—O4'	1.187 (18)
C12—H12B	0.9700	N5—O6'	1.19 (3)
C13—C14	1.513 (3)		
			
C3—O1—N1	107.05 (18)	O2—C16—N3	119.3 (2)
C1—N1—O1	107.3 (2)	O2—C16—C15	124.6 (2)
C12—N2—C8	110.77 (19)	N3—C16—C15	116.0 (2)
C12—N2—C13	110.04 (18)	N4—C17—N3	122.7 (2)
C8—N2—C13	112.10 (18)	N4—C17—C20	118.0 (2)
C12—N2—H2	107.9	N3—C17—C20	119.3 (2)
C8—N2—H2	107.9	C15—C18—N4	122.0 (2)
C13—N2—H2	108.0	C15—C18—C19	123.0 (2)
C17—N3—C16	120.4 (2)	N4—C18—C19	115.0 (2)
C17—N3—C23	124.3 (2)	C18—C19—H19A	109.5
C16—N3—C23	115.3 (2)	C18—C19—H19B	109.5
C17—N4—C18	119.8 (2)	H19A—C19—H19B	109.5
C17—N4—H4A	120.0	C18—C19—H19C	109.5
C18—N4—H4A	120.2	H19A—C19—H19C	109.5
N1—C1—C2	111.2 (2)	H19B—C19—H19C	109.5
N1—C1—C10	120.3 (2)	O3'—C20—C21'	124.6 (12)
C2—C1—C10	128.5 (2)	O3'—C20—O3	89.1 (9)
C3—C2—C7	119.3 (2)	C21'—C20—O3	79.1 (9)
C3—C2—C1	104.2 (2)	O3'—C20—C17	116.7 (9)
C7—C2—C1	136.5 (2)	C21'—C20—C17	118.3 (8)
O1—C3—C2	110.2 (2)	O3—C20—C17	108.9 (3)
O1—C3—C4	125.8 (2)	O3'—C20—C21	115.4 (9)
C2—C3—C4	124.1 (3)	O3—C20—C21	114.9 (3)
C5—C4—C3	114.4 (2)	C17—C20—C21	110.2 (3)
C5—C4—H4	122.8	C21'—C20—H20	128.1
C3—C4—H4	122.8	O3—C20—H20	108.0
F1—C5—C4	117.6 (2)	C17—C20—H20	107.9
F1—C5—C6	117.6 (3)	C21—C20—H20	106.6
C4—C5—C6	124.8 (3)	O3'—C20—H20'	105.5
C7—C6—C5	119.1 (3)	C21'—C20—H20'	67.8
C7—C6—H6	120.4	C17—C20—H20'	102.8
C5—C6—H6	120.4	C21—C20—H20'	104.4
C6—C7—C2	118.3 (2)	H20—C20—H20'	124.4
C6—C7—H7	120.8	C22—C21—C20	109.3 (3)
C2—C7—H7	120.8	C22—C21—H21A	109.8
N2—C8—C9	111.82 (19)	C20—C21—H21A	109.8
N2—C8—H8A	109.3	C22—C21—H21B	109.8
C9—C8—H8A	109.3	C20—C21—H21B	109.8
N2—C8—H8B	109.3	H21A—C21—H21B	108.3
C9—C8—H8B	109.3	C20—C21'—C22	118.7 (13)
H8A—C8—H8B	107.9	C22—C21'—H20'	128.7
C8—C9—C10	111.6 (2)	C20—C21'—H21C	107.6
C8—C9—H9A	109.3	C22—C21'—H21C	107.6
C10—C9—H9A	109.3	H20'—C21'—H21C	123.3
C8—C9—H9B	109.3	C20—C21'—H21D	107.6
C10—C9—H9B	109.3	C22—C21'—H21D	107.6
H9A—C9—H9B	108.0	H20'—C21'—H21D	65.3
C1—C10—C11	113.1 (2)	H21C—C21'—H21D	107.1
C1—C10—C9	110.9 (2)	C21—C22—C23	110.3 (3)
C11—C10—C9	108.7 (2)	C21'—C22—C23	121.7 (7)
C1—C10—H10	108.0	C21—C22—H22A	109.6
C11—C10—H10	108.0	C21'—C22—H22A	125.3
C9—C10—H10	108.0	C23—C22—H22A	109.6
C12—C11—C10	112.3 (2)	C21—C22—H22B	109.6
C12—C11—H11A	109.1	C21'—C22—H22B	72.8
C10—C11—H11A	109.1	C23—C22—H22B	109.6
C12—C11—H11B	109.1	H22A—C22—H22B	108.1
C10—C11—H11B	109.1	C22—C23—N3	113.4 (3)
H11A—C11—H11B	107.9	C22—C23—H23A	108.9
N2—C12—C11	111.7 (2)	N3—C23—H23A	108.9
N2—C12—H12A	109.3	C22—C23—H23B	108.9
C11—C12—H12A	109.3	N3—C23—H23B	108.9
N2—C12—H12B	109.3	H23A—C23—H23B	107.7
C11—C12—H12B	109.3	C20—O3—H3	109.5
H12A—C12—H12B	107.9	H20'—O3—H3	140.8
N2—C13—C14	114.5 (2)	C20—O3'—H20	49.0
N2—C13—H13A	108.6	C20—O3'—H3'	110.7
C14—C13—H13A	108.6	H20—O3'—H3'	63.4
N2—C13—H13B	108.6	O5'—N5—O4'	120.8 (15)
C14—C13—H13B	108.6	O5'—N5—O4	108.4 (14)
H13A—C13—H13B	107.6	O4'—N5—O4	130.7 (12)
C15—C14—C13	109.0 (2)	O4'—N5—O6	106.5 (11)
C15—C14—H14A	109.9	O4—N5—O6	120.5 (8)
C13—C14—H14A	109.9	O5'—N5—O5	127.9 (14)
C15—C14—H14B	109.9	O4—N5—O5	121.8 (9)
C13—C14—H14B	109.9	O6—N5—O5	117.1 (7)
H14A—C14—H14B	108.3	O5'—N5—O6'	122 (2)
C18—C15—C16	118.8 (2)	O4'—N5—O6'	114 (2)
C18—C15—C14	125.0 (2)	O6—N5—O6'	139.3 (18)
C16—C15—C14	116.1 (2)	O5—N5—O6'	103.5 (19)
			
C3—O1—N1—C1	0.0 (3)	C17—N3—C16—C15	0.0 (3)
O1—N1—C1—C2	0.7 (3)	C23—N3—C16—C15	179.0 (2)
O1—N1—C1—C10	−176.8 (2)	C18—C15—C16—O2	−176.2 (3)
N1—C1—C2—C3	−1.2 (3)	C14—C15—C16—O2	1.2 (4)
C10—C1—C2—C3	176.1 (2)	C18—C15—C16—N3	3.4 (4)
N1—C1—C2—C7	178.0 (3)	C14—C15—C16—N3	−179.2 (2)
C10—C1—C2—C7	−4.7 (5)	C18—N4—C17—N3	2.0 (4)
N1—O1—C3—C2	−0.8 (3)	C18—N4—C17—C20	−176.8 (2)
N1—O1—C3—C4	178.5 (2)	C16—N3—C17—N4	−2.8 (4)
C7—C2—C3—O1	−178.2 (2)	C23—N3—C17—N4	178.3 (2)
C1—C2—C3—O1	1.2 (3)	C16—N3—C17—C20	176.0 (2)
C7—C2—C3—C4	2.5 (4)	C23—N3—C17—C20	−2.8 (4)
C1—C2—C3—C4	−178.1 (2)	C16—C15—C18—N4	−4.4 (4)
O1—C3—C4—C5	179.9 (2)	C14—C15—C18—N4	178.4 (2)
C2—C3—C4—C5	−0.9 (4)	C16—C15—C18—C19	176.1 (2)
C3—C4—C5—F1	177.3 (2)	C14—C15—C18—C19	−1.1 (4)
C3—C4—C5—C6	−1.6 (4)	C17—N4—C18—C15	1.7 (4)
F1—C5—C6—C7	−176.4 (2)	C17—N4—C18—C19	−178.7 (2)
C4—C5—C6—C7	2.4 (4)	N4—C17—C20—O3'	25.3 (10)
C5—C6—C7—C2	−0.7 (4)	N3—C17—C20—O3'	−153.5 (10)
C3—C2—C7—C6	−1.6 (4)	N4—C17—C20—C21'	−161.0 (11)
C1—C2—C7—C6	179.3 (3)	N3—C17—C20—C21'	20.1 (12)
C12—N2—C8—C9	−55.1 (3)	N4—C17—C20—O3	−73.5 (3)
C13—N2—C8—C9	−178.4 (2)	N3—C17—C20—O3	107.6 (3)
N2—C8—C9—C10	56.2 (3)	N4—C17—C20—C21	159.6 (3)
N1—C1—C10—C11	−17.7 (3)	N3—C17—C20—C21	−19.3 (4)
C2—C1—C10—C11	165.2 (2)	O3'—C20—C21—C22	−171.6 (10)
N1—C1—C10—C9	104.7 (3)	C21'—C20—C21—C22	−57.1 (13)
C2—C1—C10—C9	−72.4 (3)	O3—C20—C21—C22	−70.0 (4)
C8—C9—C10—C1	180.0 (2)	C17—C20—C21—C22	53.5 (4)
C8—C9—C10—C11	−55.1 (3)	O3'—C20—C21'—C22	148.9 (15)
C1—C10—C11—C12	179.3 (2)	O3—C20—C21'—C22	−129.9 (17)
C9—C10—C11—C12	55.7 (3)	C17—C20—C21'—C22	−24 (2)
C8—N2—C12—C11	55.1 (3)	C21—C20—C21'—C22	62.1 (14)
C13—N2—C12—C11	179.6 (2)	C20—C21—C22—C21'	49.6 (12)
C10—C11—C12—N2	−56.7 (3)	C20—C21—C22—C23	−66.4 (4)
C12—N2—C13—C14	163.5 (2)	C20—C21'—C22—C21	−69.2 (16)
C8—N2—C13—C14	−72.8 (3)	C20—C21'—C22—C23	13 (2)
N2—C13—C14—C15	167.7 (2)	C21—C22—C23—N3	43.2 (4)
C13—C14—C15—C18	89.9 (3)	C21'—C22—C23—N3	3.9 (12)
C13—C14—C15—C16	−87.4 (3)	C17—N3—C23—C22	−8.4 (4)
C17—N3—C16—O2	179.7 (2)	C16—N3—C23—C22	172.7 (3)
C23—N3—C16—O2	−1.4 (4)		

### Hydrogen-bond geometry (Å, º)

**Table:**

*D*—H···*A*	*D*—H	H···*A*	*D*···*A*	*D*—H···*A*
N2—H2···O4′	0.91	1.94	2.823 (18)	164
N2—H2···O5	0.91	2.15	3.028 (12)	161
N2—H2···O6	0.91	2.23	2.999 (9)	142
N2—H2···N5	0.91	2.56	3.465 (3)	170
N2—H2···O5′	0.91	2.58	3.36 (3)	144
O3′—H3′···N1^i^	0.82	2.30	3.117 (18)	169
O3—H3···O5^i^	0.82	2.11	2.915 (12)	168
O3—H3···O4′^i^	0.82	2.27	3.064 (19)	163
O3—H3···O6′^i^	0.82	2.30	2.93 (4)	134
O3—H3···O4^i^	0.82	2.60	3.187 (16)	130
O3—H3···N5^i^	0.82	2.67	3.423 (4)	153

Symmetry code: (i) −*x*+1, *y*−1/2, −*z*+3/2.
